# Fennel Essential Oil as a Complementary Therapy in the Management of Diabetes

**DOI:** 10.3390/pharmaceutics15122657

**Published:** 2023-11-23

**Authors:** Ruxandra Ștefănescu, Bianca-Eugenia Ősz, Andrada Pintea, Eszter Laczkó-Zöld, Amelia Tero-Vescan, Camil-Eugen Vari, Emoke Fulop, Iuliana Blaș, Szende Vancea

**Affiliations:** 1 Department of Pharmacognosy and Phytotherapy, Faculty of Pharmacy, George Emil Palade University of Medicine, Pharmacy, Science and Technology of Târgu Mures, 38 Gheorghe Marinescu Street, 540139 Târgu Mures, Romania; ruxandra.stefanescu@umfst.ro (R.Ș.); eszter.laczko@umfst.ro (E.L.-Z.); 2 Department of Pharmacology and Clinical Pharmacy, Faculty of Pharmacy, George Emil Palade University of Medicine, Pharmacy, Science and Technology of Târgu Mureș, 38 Gheorghe Marinescu Street, 540139 Târgu Mures, Romania; camil.vari@umfst.ro; 3 Faculty of Pharmacy, George Emil Palade University of Medicine, Pharmacy, Science and Technology of Târgu Mureș, 38 Gheorghe Marinescu Street, 540139 Târgu Mures, Romania; andradapintea@yahoo.com (A.P.); iuliana867@gmail.com (I.B.); 4 Department of Biochemistry, Faculty of Medicine, George Emil Palade University of Medicine, Pharmacy, Science and Technology of Târgu Mureș, 38 Gheorghe Marinescu Street, 540139 Târgu Mures, Romania; amelia.tero-vescan@umfst.ro; 5 Emergency County Hospital Târgu Mureș, Department of Pathology, 50 Gh. Marinescu Street, 540136 Târgu Mureș, Romania; emifulop@yahoo.com; 6 Legal Medicine Service, Emergency County Hospital Miercurea Ciuc, 530173 Miercurea Ciuc, Romania; vancsa.szende@gmail.com

**Keywords:** diabetes, fennel essential oil, aromatherapy

## Abstract

Diabetes is a serious pathology that affects a significant number of people worldwide. Its progression is rapid and leads to serious complications if glycemic control is missing. The micro and macrovascular complications of diabetes produce disabilities over time that affect the daily lives of patients. The major challenge of diabetes therapy is to reach a stable glycemic state and to delay the onset of specific complications. Aromatherapy is considered an alternative or complementary therapy, but in recent years, there has been a tendency to overuse essential oils. The present study was designed to evaluate and compare the effects produced by the topical and oral administration of fennel essential oil to diabetic rats. Eighteen compounds in fennel essential oil were identified by gas chromatography–mass spectrometry (GC-MS) analysis. The major compounds were *trans*-anethole (64.6%) and fenchone (24.5%). The in vivo study revealed that after a four-week treatment with fennel essential oil, the rats’ glycemic levels were significantly reduced (*p* ≤ 0.05). Furthermore, there were no differences between the two routes of administration. In addition, an ex vivo study underlined the potential effect of this essential oil in the prevention of cataract formation.

## 1. Introduction

Diabetes has become a worldwide health issue with an unfavorable long-term prognosis. The global prevalence of diabetes has increased rapidly over the last decade, and its rising trend in developing countries is alarming and is probably due to a decrease in food quality and modifications in eating behaviors [1]. Uncontrolled diabetes has the potential to cause serious complications, such as neuropathy, nephropathy, retinopathy, cataracts, and sexual dysfunction, with irreversible symptoms that consequently lead to high morbidity and high mortality [2,3]. 

With the progress in pharmacology, a wide range of therapeutic options are available, but still, the management of diabetes and diabetic complications is difficult due to the sometimes unpredictable response to treatment. Also, patient compliance, related or not to the side effects of the treatment, greatly influences the treatment outcome, so therapeutic failure is a common problem. In addition to all of these difficulties, the management of diabetes in developing countries encounters other problems, such as reduced accessibility to treatment and even lower compliance with treatment; therefore, a large number of patients do not achieve the treatment goals.

Alongside the progress in pharmacology, progress in phytopharmacology revealed that natural compounds could be an option in the management of diabetes, either included in the therapeutic scheme as a complementary treatment, with the possibility of reducing the dose of the synthetic drug, or as an alternative treatment when synthetic options are unavailable (as in many developing countries). Natural compounds belonging to different chemical classes (polyphenolic compounds, curcumin, and sterolic saponins) were evaluated, and some of them showed promising therapeutic potential [3,4,5].

Aromatherapy has gained a lot of interest in recent years, and the overwhelming majority of empirical proof raises the need for the pharmacological evaluation of essential oils. The “essential’’ question that still arises is whether the pharmacokinetic profile of volatile compounds justifies the internal administration of essential oils (which comes with serious risks), or whether the same pharmacological effects can be obtained by topical administration and/or inhalation [6,7]. 

Foeniculi fructus is the fruit harvested from the species Foeniculum vulgare Mill., Apiaceae family, also called sweet anise, large anise, German anise, badian, garden-sweet cumin, and fennel. These fruits have a long history of medicinal use, especially as a carminative and galactagogue. Moreover, different studies suggest that this herbal drug has a plethora of effects: antibacterial, antioxidant, anti-inflammatory, hepatoprotective, etc. [8]. According to the European Medicines Agency (EMA), the fruits are indicated as carminatives and expectorants [9]. The fruits contain volatile components as well as non-volatile compounds. The non-volatile compounds identified in fennel fruits are mainly phenolic compounds like hydroxycinnamic acid derivatives and flavonoid derivatives [10]. The volatile compounds that are dominant in the essential oil are trans-anethole, estragole, and fenchone. Their proportion varies depending on their geographic origin [11,12]. Until now, only a few research studies have assessed the effects of fennel products on diabetes. Most studies were performed in vitro, but preclinical studies have evaluated the effects of different extracts from fennel on diabetic rats [13,14,15]. While the in vitro studies have shown promising effects, information from in vivo studies is scarce. 

The present study aimed to compare the effects of oral administration of fennel essential oil (FEO) or the topical application of FEO in rats with streptozotocin-induced diabetes. The present study also evaluated FEO’s ex vivo anticataractogenic effect. 

## 2. Materials and Methods

### 2.1. Plant Material

Fruits of *Foeniculum vulgare* Mill. sp. *vulgare*, var. *vulgare* were harvested in August 2017 from the Garden of Medicinal Plants of the George Emil Palade University of Medicine, Pharmacy, Science and Technology of Târgu Mureș (46°32′18.9096″ N, 24°33′5.1804″ E). The essential oil was obtained according to the method described in the 10th edition of the European Pharmacopoeia, using a Neo-Clevenger type distillation apparatus (Glassco, Manglai, India) [16]. A voucher specimen, codified FVGPM-1704, of the herbal drug was deposited in the herbarium of the discipline of Pharmacognosy and Phytotherapy from George Emil Palade University of Medicine, Pharmacy, Science, and Technology of Târgu Mureș.

### 2.2. Reagents

Streptozotocin (STZ), DL-glyceraldehyde, and analytical-grade solvents were purchased from Sigma–Aldrich (St. Louis, MO, USA). NADPH tetrasodium salt and 2-mercaptoethanol were purchased from Carl Roth GmbH (Karlsruhe, Germany).

### 2.3. GC-MS Quantification of Volatile Compounds

The gas chromatography–mass spectrometry (GC-MS) instrument was a 7890B GC-5977A MSD system (Agilent Technologies, Santa Clara, CA, USA) equipped with an HP-5MS UI, 30 m × 0.25 mm, 0.25 μm (Agilent Technologies) column. Helium was used as the carrier gas with a flow rate of 1 mL/min. The oven program: 60 °C (1 min), 60–250 °C (ramp 10 °C/min), hold 250 °C (5 min). Spitless injection, 1 μL sample volume injected into the column. MS detection parameters: scan mode, in the range of 40–500 *m*/*z*. MS spectra analysis was performed using NIST14 and MPW5e (version 14) (Wiley) libraries. Sample preparation: essential oil samples were diluted 400-fold with hexane (GC-MS purity).

### 2.4. Animals

The experimental study on laboratory animals was carried out with the consent of the Ethics Commission of the University (Registration number: 50-10/04/2017). Female Wistar white rats with a weight of 270 ± 14 g were provided by the University’s Biobase. Before starting the experiment, the animals were kept for one week under standard breeding conditions (12-h day/night cycle, access to food and water ad libitum) to adapt to the laboratory conditions.

### 2.5. Experimental Protocol

Diabetes was induced by intraperitoneal injection with streptozotocin (2-deoxy-2,3-methyl-nitrozoureido-*p*-glucopyranose) (STZ), 60 mg/kg body weight, after a 12-h fasting period. Immediately after the STZ injection, the rats had free access to food and water to prevent second-stage deaths from the injection due to the destruction of beta-pancreatic cells and a massive release of insulin by the pancreas [17].

Fasting blood glucose (FBG) was determined 48 h after the injection using a commercial glucometer (AccuCheck Active, Roche Diabetes Care Inc., Indianapolis, IN, USA) with blood collected from the tail vein; diabetic rats were considered to be those with glycemia ≥ 250 mg/dL.

The rats were divided after simple randomization into four groups, 8 rats/group, as follows: DC—diabetic control, DM—diabetic rats treated with 300 mg/kg metformin, DFO—diabetic rats treated orally with 400 mg/kg fennel essential oil, DFT—diabetic rats treated topically with 400 mg/kg fennel essential oil. 

Body weight was measured at the beginning of the study before the induction of diabetes and at the end of the experiment. FBG was determined weekly from the tail vein, following a sterile pinprick of the distal tail vein.

In the 4th week of the experiment, an oral glucose tolerance test (OGTT) was performed after a 12-h fast. Each rat was loaded with a dose of 2 g/kg body mass of sterile 50% (*w*/*v*) D-glucose solution via orogastric gavage. The blood glucose values were determined at 0, 30, 60, 90, and 120 min for each animal.

Aria under the curve (AUC) for the glucose level over time (0–120 min) was calculated using a linear trapezoidal method (Equation (1)):

(1) $${AUC}_{0–120=}\frac{\left(C_{0}+C_{30})\;\times \;(t_{30}-t_{0}\right)}{2}+\frac{\left(C_{30}+C_{60})\;\times \;(t_{60}-t_{30}\right)}{2}+\frac{\left(C_{60}+C_{90})\;\times \;(t_{90}-t_{60}\right)}{2}+\frac{\left(C_{90}+C_{120})\;\times \;(t_{120}-t_{90}\right)}{2}$$

where *AUC* represents the area under the curve, *C*_0_, *C*_30_, *C*_60_, *C*_90_, and *C*_120_ are the blood glucose levels in mg/dL at different times, and *t*_0_, *t*_30_, *t*_60_, *t*_90_, and *t*_120_ are the time values in minutes.

At the end of the experiment, the rats were euthanized with isoflurane, and the kidneys, liver, and eyes were collected. 

### 2.6. Histopathological Analysis

Livers from 4 rats/group were collected and immediately placed in 10% formalin. Three different types of staining were performed using hematoxylin-eosin (H&E), periodic acid Schiff (PAS), and von Gyeson reagents.

### 2.7. Ex Vivo Anticataractogenic Effect Evaluation

Immediately after the removal of the eyes, the lenses were isolated by retro-orbital incision and were introduced into saline solution. The determination was performed using sterile Petri dishes, and Roti @ CELL-Medium (with glutamine and HEPES) was used. Glucose at a concentration of 55 mM was used to induce opacification. A total of 12 lenses were used, which were divided into 3 groups: 1. NC—Normal control: culture medium; 2. C—Control: culture medium + 55 mM glucose; 3. F–Fennel: culture medium + 55 mM glucose + fennel essential oil. The samples were incubated for 72 h at 37 °C, and the medium was changed daily. At the end of the experiment, the degree of opacification was evaluated under a microscope based on a scale from 0 to 3, previously described in the literature, as follows: • Grade 0—crystal clear, absence of opacification; • Grade 1—slight degree of opacification, weakly blurred appearance; • Grade 2—diffuse opacification, which includes almost the entire lens; • Grade 3—marked opacification of the entire lens [18,19].

### 2.8. Statistical Analysis

Statistical analysis was performed using GraphPad Prism 9.0 software. Results were expressed as mean ± standard deviation. For the in vivo study, the comparisons between groups were performed using the ANOVA variance test and the Tukey–Kramer post hoc test. The Kruskal–Wallis test followed by the Dunn test was used to compare the degree of opacification between groups from the ex vivo study.

## 3. Results

The essential oil concentration of the fruits of *Foeniculum vulgare* harvested from the Garden of Medicinal Plants of the University of Medicine and Pharmacy of Târgu Mureș was 4.96 ± 0.15%. This concentration is in accordance with the requirements of the European Pharmacopoeia 10th edition, which recommends a concentration of a min. 4% essential oil in the fruits [16].

### 3.1. GC-MS Analysis of Fennel Essential Oil

**Figure 1:**
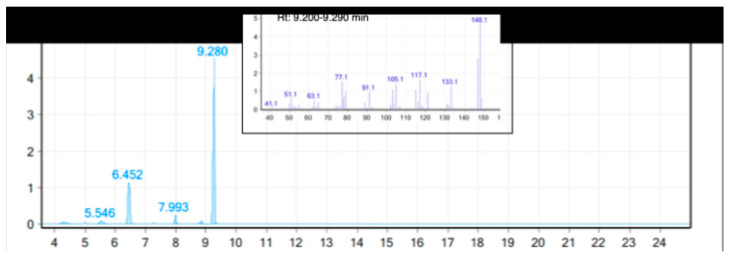
GC-MS chromatogram of the diluted fennel essential oil components. Peaks are numbered with their retention time.

The GC-MS analysis revealed the presence of eighteen volatile compounds, with trans-anethole and fenchone being the dominant compounds (Figure 1). As can be observed in Table 1, the composition of the essential oil used in this study is in accordance with the Eur. Ph. 10th edition [16].

The relative concentration of each identified compound was calculated as the peak area to the total area of all identified peaks.

### 3.2. In Vivo Study

At 48 h following the STZ injection, the rats displayed diabetes-related symptoms like polydipsia and polyuria, observed through excessive water consumption and litter wetting—phenomena that were not present during the animals’ initial week of acclimation.

#### 3.2.1. Weight and Glucose Variations during the In Vivo Study

In the second week of treatment (after the STZ injection), a decrease in blood glucose level was observed, but with the progression of diabetes, in the second and third week of treatment, there were high blood glucose levels. After starting the treatment in the metformin-treated group (DM), blood glucose levels remained constant, with no statistically significant differences from baseline (measured before diabetes was induced). In the group treated orally with FEO, a reduction in the glucose level was observed in the second week of treatment. The difference from the value of glycemia in the first week was statistically significant. Although the blood glucose levels were lower in week 3 and 4 than in week 1, the differences were not statistically significant. The animals in the DFT group had the same outcome.

**Figure 2:**
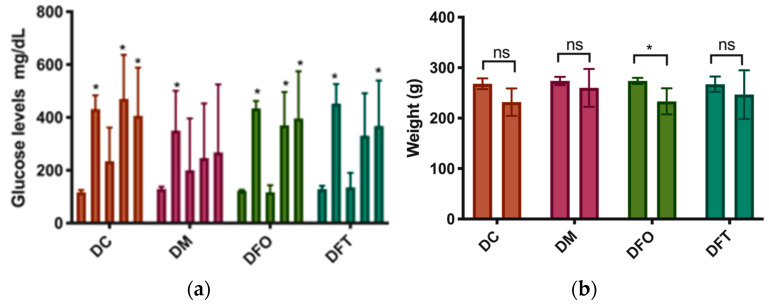
(**a**) Glucose level mean ± SD in every week, starting with 0 (prior to STZ induction), week 1 (48 h after STZ administration), week 2, week 3, and week 4. *—statistically significant difference in the same group (*p* < 0.05); (**b**) Mean weight in the first week of the experiment and the final week. The results are expressed as means ± SD; ns—not significant, *—statistically significant difference in the first week compared with the final week (*p* < 0.05).

As can be seen in Figure 2, in the second week of treatment, the lowest blood glucose values were recorded in all of the study groups. Regarding the rats’ weight, comparing the average weight of the rats in each group at the end of the experiment with the initial average, a decrease in weight was observed in all groups as expected due to the type of diabetes. Following the statistical analysis, it was observed that the weight loss was significantly lower at the end of the experiment only in the group treated orally with FEO, as can be seen in Figure 2.

#### 3.2.2. Oral Glucose Tolerance Test (OGTT)

**Figure 3:**
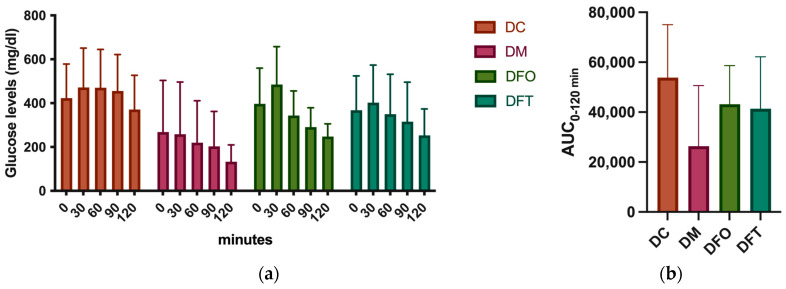
OGTT results. (**a**) Glucose levels at 0, 30, 60, 90, and 120 min; (**b**) AUC_0–120 min_, DC—diabetic control, DM—diabetic rats treated with 300 mg/kg metformin, DFO—diabetic rats treated orally with 400 mg/kg fennel essential oil, DFT—diabetic rats treated topically with 400 mg/kg fennel essential oil. ANOVA analysis indicated that there were no statistically significant differences in the same group for the OGTT test (*p* ≤ 0.05).

During the oral glucose tolerance test, a marked increase in blood glucose was observed in the first phase (Figure 3). After 60 min, a decrease in blood glucose was observed in the groups that received treatment. The most marked decrease was observed in the group treated with metformin, followed by the group treated orally with fennel essential oil.

#### 3.2.3. Histopathological Analysis of the Liver

**Figure 4:**
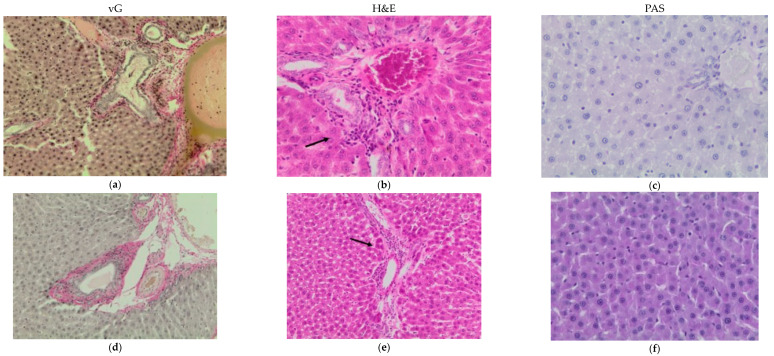

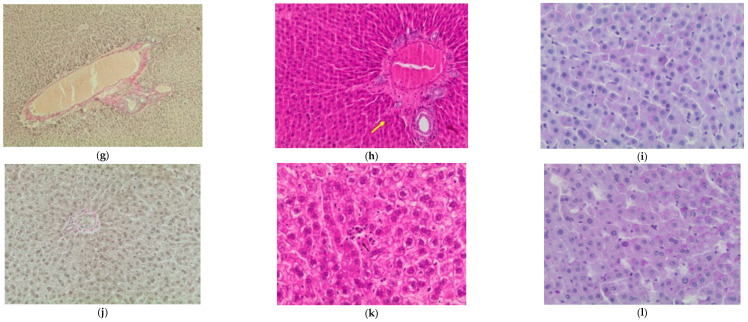
Liver histopathological analysis with van Gieson, H&E, and PAS staining. (**a**–**c**) Diabetic control group: severe fibrosis, mononuclear inflammatory infiltrate, low glycogen; (**d**–**f**) Diabetic group treated with metformin: fibrosis in the portal area, portal inflammatory infiltrate (black arrow); (**g**–**i**) Diabetic group treated with fennel essential oil oral: minor signs of fibrosis, biliary duct hyperplasia (yellow arrow); (**j**–**l**) Diabetic group treated with fennel essential oil topical: no or minor signs of fibrosis, inflammation in sinusoids (black arrow).

Histopathology of the liver showed periportal inflammation and fibrosis, severe degeneration, and a reduction in glycogen. Areas of chronic inflammation with eosinophil infiltration were seen in all groups, but the intensity was lower in the FEO group. Also, after PAS staining, low glycogen could be observed in the diabetic control group (Figure 4).

### 3.3. Ex Vivo Study

**Figure 5:**
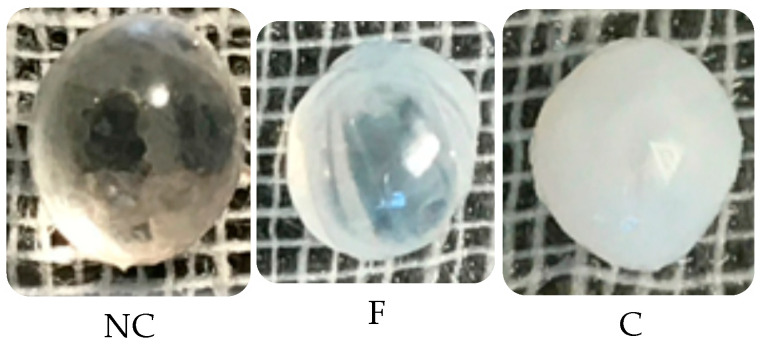
Lenses at the end of the ex vivo study; NC—normal control (lenses in culture medium), grade 0, C—control (culture medium + 55 mM glucose), grade 3, F—fennel essential oil treated (culture medium + 55 mM glucose + fennel essential oil), grade 1.

Statistical analysis showed that there were no statistical differences in group NC and group F (*p* ≤ 0.05). The lenses in the positive control group showed a complete loss of transparency (Grade 4) confirmed by the invisibility of the grid lines (Figure 5).

## 4. Discussion

Fennel essential oil can be obtained by hydro distillation or by steam distillation. The oil has an aromatic smell, a slight yellowish color, and a sweet or bitter taste, depending on the variety of *Foeniculum vulgare* used for distillation. The main compound found in the essential oil is anethole, and depending on the variety, fenchone can be found in different concentrations. According to the European Pharmacopoeia, the oil obtained from the fruits harvested from *Foeniculum vulgare* Miller sp. *vulgare* var. *vulgare* must contain a minimum 60% anethole and 15% fenchone and a maximum 6% estragole, while the essential oil obtained from *Foeniculum vulgare* Miller sp. *vulgare* var. *dulce* (Miller) Thellung must contain a minimum of 80% anethole and a maximum 7.5% fenchone and 10% estragole [16]. Due to the estragole content, the HMPC’s (The Committee on Herbal Medicinal Products) from EMA (European Medicines Agency) final opinion was that the European Union’s herbal monograph on *Foeniculum vulgare* Miller subsp. *vulgare* var. *vulgare*, aetheroleum, cannot be supported anymore [20]. This conclusion was drawn due to the recent data related to the genotoxicity and carcinogenicity of estragole. The European Pharmacopoeia admits a maximum content of 6% estragole, but few essential oils producing companies analyze their oils and report the results in accordance with the European Pharmacopoeia [16]. Our results are in accordance with the European Pharmacopoeia and with the research conducted by Ghasemian et al., showing that trans-anethole and fenchone are the quantitatively predominant compounds [12]. 

The number of animals used in this study was chosen by taking into account the use of a minimum number of animals. To reduce the number of animals used in this study, each batch served as its control, so the final results were compared with the determinations made at the beginning of the study, before the induction of diabetes. Forty-eight hours after the STZ injection, the rats showed diabetes-specific symptoms like polydipsia and polyuria, symptoms that were lacking in the first week of accommodation. The dose of essential oil that was administered both topically and orally was chosen to take into account the LD_50_ (lethal dose 50) reported in the literature: 3.8 g/kg for oral administration and >5 g/kg for topical administration [20,21].

No toxicity symptoms like respiratory distress, prostration, sedation, movement disorders, etc., were noticed during this study. This experimental model of diabetes is similar to human type I diabetes and has become a reliable model in the investigation of diabetes therapy like the antihyperglycemic effect of various plant extracts or products. 

No deaths were reported in any of the groups during the study. Usually, deaths are rarely reported in the literature for this type of study, but it has been observed that a mortality rate of 20% is normal in this animal model [22]. The lack of mortality in the experiment is probably normal, considering that it was a short-term study and the specific complications of diabetes that can lead to death develop over a more extended period of time.

By topical administration, the essential oil quickly reaches the bloodstream due to rats’ rich vascularity and their thin layer of skin. As a result, topical administration is a preferred method for delivering a wide array of volatile compounds. A comparison between the group treated orally with fennel essential oil and the group treated topically revealed only minor differences in their outcomes, according to our study. These observations suggest that the positive effects of fennel essential oil (FEO) can be achieved through topical application with a similar efficacy to oral ingestion. This route of administration minimizes the potential side effects that might appear after oral administration. The positive effects of fennel products on diabetes are attributed to anethole, which is the quantitatively dominant component in fennel essential oil. Vellapandian et al. have previously demonstrated that the administration of anethole-rich extracts can ameliorate renal damage in diabetic rats at a dose of 100 mg anethole/kg [23,24]. Similar results have been obtained by Samadi-Hoshahr et al., at a dose of 80 mg anethole/kg [25]. The findings reported by the mentioned authors provide evidence that anethole holds significant promise in the context of diabetes pathology, suggesting it has the ability to modulate key pathways. The anti-inflammatory, antioxidant, and anti-apoptotic properties of anethole can be used to explain why it has a positive impact on renal function. The administration of the natural compound stimulates antioxidant mechanisms, increasing reduced glutathione (GSH) levels and superoxide dismutase (SOD), catalase (CAT), and glutathione peroxidase (GPx) activity, and decreasing malondialdehyde (MDA), a marker of lipid peroxidation. These effects have been observed in preclinical studies. In addition, alterations in caspases 3 and 9, monocyte chemoattractant protein-1 (MCP-1), tumor necrosis factor alpha (TNF-alpha), interferon gamma (IFN-gamma), and interleukin 10 (IL-10) levels suggest anti-inflammatory and anti-apoptotic effects [26]. In addition to these effects, the ability of anethole to block the activity of angiotensin-converting enzyme in a dose-dependent manner was also observed [27]. This mechanism, in turn, can contribute to its reno-protective action by normalizing renal function as a result of the decrease in the level of angiotensin II (AT-II), a vasoconstrictor substance at the renal level, an effect mediated by AT1 receptors (AT1R). Precisely for these reasons, it is recommended for diabetic patients to take substances that inhibit the synthesis of ATII (angiotensin-converting enzyme inhibitors, ACEI) or prevent the action of ATII by directly blocking AT1R (angiotensin-receptor blockers, ARBs) [28]. But, taking into account the fact that AT-II increases the tissue expression of transforming growth factor beta-1 (TGF-β1), a profibrotic growth factor, the beneficial effect of anethole could also be explained based on this mechanism [29]. Specifically, TGF-β1-induced fibrosis occurs as a result of activation of the SMAD signaling pathway. Phosphorylation of SMAD proteins, specifically SMAD 3 and SMAD 4, contributes to increased collagen synthesis and favors fibrosis [30].

Inhibition of angiotensin-converting enzyme activity could also explain the hepatoprotective action of anethole. Previous studies suggested that AT-II induces the proliferation of hepatic stellate cells and increases the expression of the messenger RNA (mRNA) that encodes transforming growth factor beta-1 (TGF-β1). This effect occurs as a result of the agonistic action of ATII on AT1R receptors. In the same study, it was shown that the administration of an ACEI (perindopril) and an ARB (candesartan) would have a protective effect on the evolution of liver fibrosis [31]. As observed in the histopathological analysis in this study, metformin treatment showed some improvements in fibrosis and glycogen content, while both oral and topical administration of fennel essential oil appeared to have a protective effect against fibrosis but influenced glycogen storage differently. In contrast, the glycogen content in the DC group was observed to be low, possibly reflecting impaired glycogen storage due to diabetes-induced liver dysfunction. Nonetheless, the multiple beneficial effects of fennel could be the result of synergistic actions between anethole and fenchone and/or other volatile constituents. Fenchone and fenchone-rich essential oils have been shown to have antihyperglycemic effects as well as antioxidant effects in rats with alloxan-induced diabetes [32]. Fenchone’s antioxidant properties may minimize kidney and liver damage [32].

Recent studies have shown that synthetic fenchone derivatives possess an affinity for cannabinoid 2 receptors (CB2R). CB2R are predominantly located in the periphery, and their stimulation has beneficial effects in chronic inflammatory and neuropathic pain. Following CB2R stimulation, an increase in the production of IL-10, a cytokine with anti-inflammatory action, was observed [33,34,35].

The glucose AUC is a better indicator of glucose tolerance. The results indicated that the highest glucose AUC after a glucose tolerance test was recorded in the diabetic control group, while the lowest was recorded in the metformin-treated group. Although there were no significant differences between groups, due to the large variations within the same group, in the FEO group, the AUC was lower than in the control group. This prompts the thought that fennel essential oil could interfere with the enzymes involved in glycemic regulation, like α-glucosidase and α-amylase. But, since the DFT group received the treatment topically, we cannot consider such a direct mechanism of action but rather a regulatory effect on pancreatic secretion. Intracellular glucose uptake is mediated by isozymes from the hexokinase class with a different distribution in the body and is characterized by different values of the Michaelis–Menten constant (Km), reflecting different affinities. Hepatic glucokinase has the highest Km value and, therefore, the lowest affinity. It is activated by insulin and catalyzes the rate-limiting step in hepatic glucose uptake. It is considered that the binding of small molecules, such as the constituents of essential oils, to an allosteric center of the enzyme causes conformational changes of the active center, making it more capable of binding to the substrate [36]. Among the mechanisms proposed for an antidiabetic effect, in addition to an inhibitory effect on alpha-amylase and alpha-glucosidase, there is also an inhibitory effect on tyrosine-protein phosphatase non-receptor type 1 (PTP1B), the enzyme-type receptor that catalyzes the hydrolysis of phosphate monoesters specifically on tyrosine residues, thus having an anti-insulin effect [37].

One of the most significant consequences of diabetes is cataracts. This gradually worsens as diabetes progresses, especially if there are sharp fluctuations in blood sugar levels and when glycemic levels are not sustained over time. The pursuit of stronger substances that can prevent diabetic cataracts at low doses or for new aldose reductase inhibitors with no side effects is still ongoing. Aldose reductase catalyzes the reduction of glucose to sorbitol, an osmotic substance that affects the integrity of the lens by stimulating osmotic stress and epithelial cell death [38]. In case of a tissue injury, the production of reactive oxygen species (ROS) increases. Reducing the level of ROS and inflammatory mediators is achieved physiologically by activating antioxidant mechanisms (e.g., SOD). Activation of endogenous antioxidant systems and inhibition of apoptotic processes is achieved through the Nrf2 (nuclear erythroid 2-related factor) pathway [39,40].

Since *trans*-anethole has been shown to activate the Nrf2 pathway, this mechanism could explain the antioxidant and antiapoptotic effects of this natural compound, including its anti-cataract effect. In the ex vivo study, the lenses treated with fennel essential oil did not reach the 3rd grade of opacification compared with the lenses from the control group, where no treatment was added to the medium. However, since essential oils are a complex mixtures of volatile compounds, the effects observed in our study may also be due to the anti-inflammatory action of fenchone as a result of CB2R stimulation, a receptor expressed in large numbers in the eye. In the case of essential oils, as well as in other herbal extracts, it is difficult to identify exactly the compound responsible for the therapeutic effect.

## 5. Conclusions

The findings of the current investigation showed that fennel essential oil has antihyperglycemic effects in rats with streptozotocin-induced diabetes. Statistically significant effects were observed regardless of the route of oil administration (*p* ≤ 0.05). This study demonstrates that therapeutic effects can be obtained with topical application, thereby preventing a series of side effects that occur after oral administration. The ex vivo study demonstrated an anti-cataractogenic effect, but further studies are needed in order to see if these effects can be reached through topical administration. The present results add valuable contributions to the scientific literature, emphasizing the need for using essential oils with a known composition as well as the importance of using essential oils rationally in the complementary treatment of diabetes.

## Figures and Tables

**Table 1:** Composition of fennel essential oil.

Compound	Rt	Area%
Monoterpenes		
*α*-Pinene	4.315	2.25
Camphene	4.568	0.08
Sabinene	4.858	0.13
*β*-Myrcene	4.988	0.62
*α*-Phellandrene	5.233	0.15
D-Limonene	5.572	2.27
*γ*-Terpinene	5.994	0.20
Oxygenated monoterpenes		
Fenchone	6.587	24.51
(+)-2-Bornanone	7.308	0.63
1-Menthone	7.391	0.08
dl-Menthol	7.627	0.07
Terpinen-4-ol	7.732	0.13
Terpineol	7.895	0.07
Phenylpropanoids		
Estragole	8.044	2.77
Anethole	8.759	0.13
*trans*-Anethole	9.279	64.63
Other oxygenated compounds		
2-Propanone	10.582	0.11
4-Methoxy Benzaldehyde	8.844	0.98

## Data Availability

Data are contained within the article.
